# Blockade of Mas Receptor or Mas-Related G-Protein Coupled Receptor Type D Reduces Portal Pressure in Cirrhotic but Not in Non-cirrhotic Portal Hypertensive Rats

**DOI:** 10.3389/fphys.2019.01169

**Published:** 2019-09-20

**Authors:** Lakmie S. Gunarathne, Peter W. Angus, Chandana B. Herath

**Affiliations:** ^1^Department of Medicine, The University of Melbourne, Austin Health, Melbourne, VIC, Australia; ^2^Department of Gastroenterology and Hepatology, Austin Health, Melbourne, VIC, Australia

**Keywords:** portal pressure, portal hypertension, mas receptor, Mas-related G-protein coupled receptor type D, angiotensin II type 1 receptor, cirrhosis, systemic hypotension, mesenteric vasculature

## Abstract

Portal hypertension (PHT) resulting from splanchnic vasodilatation is a major cause of morbidity and mortality in patients with cirrhosis. The renin-angiotensin system (RAS) plays an important role in splanchnic vasodilatation in cirrhosis. This study investigated whether acute blockade of the vasodilatory receptors of the alternate RAS, Mas (MasR), Mas-related G-protein coupled receptor type D (MrgD), and angiotensin II type-2 receptor (AT2R) improves PHT in cirrhotic and non-cirrhotic portal hypertensive rats and counteracts systemic hypotension associated with angiotensin II type 1 receptor (AT1R) blockade. Cirrhotic bile duct ligated (BDL) or carbon tetrachloride (CCl_4_) injected and non-cirrhotic partial portal vein ligated (PPVL) rats were used for measurement of portal pressure (PP) and mean arterial pressure before and after an intravenous bolus injection of the MasR, MrgD, and AT2R blockers, A779, D-Pro^7^-Ang-(1-7) (D-Pro) and PD123319, respectively. Separate groups of rats received a combined treatment with A779 or D-Pro given 20 min after AT1R blocker losartan. Mesenteric expression of MasR, MrgD, and AT2R and circulating levels of peptide blockers were also measured. Treatment with A779 and D-Pro significantly reduced PP in cirrhotic rat models. Despite rapid degradation of A779 and D-Pro in the rat circulation, the PP lowering effect of the blockers lasted for up to 25 min. We also found that PD123319 reduced PP in CCl_4_ rats, possibly by blocking the MasR and/or MrgD since AT2R expression in cirrhotic mesenteric vessels was undetectable, whereas the expression of MasR and MrgD was markedly elevated. While losartan resulted in a marked reduction in PP, its profound systemic hypotensive effect was not counteracted by the combination therapy with A779 or D-Pro. In marked contrast, none of the receptor blockers had any effect on PP in non-cirrhotic PPVL rats whose mesenteric expression of MasR and MrgD was unchanged. We conclude that in addition to MasR, MrgD, a newly discovered receptor for Angiotensin-(1-7), plays a key role in splanchnic vasodilatation in cirrhosis. This implies that both MasR and MrgD are potential therapeutic targets to treat PHT in cirrhotic patients. We also conclude that the alternate RAS may not contribute to the development of splanchnic vasodilatation in non-cirrhotic PHT.

## Introduction

Portal hypertension (PHT) is a clinical syndrome defined by a pathological increase of pressure within the portal vascular system and is the major cause of morbidity and mortality in patients with cirrhosis (Rodriguez-Vilarrupla et al., 2007). Portal hypertension results from both increased hepatic resistance due to fixed obstruction of portal flow and active contraction of activated stellate cells and vascular smooth muscle cells (Bataller and Brenner, 2005; Di Pascoli et al., 2017) and increased portal inflow due to pathological vasodilatation of the splanchnic vascular bed, a consequence of the hyperdynamic circulation secondary to liver cirrhosis (Vorobioff et al., 1983; Bolognesi et al., 2014; Di Pascoli et al., 2017).

The main therapy used to prevent variceal bleeding in cirrhotic patients with PHT is non-selective β-blockade (NSBB), which reduces portal pressure by decreasing splanchnic blood flow and increasing mesenteric tone (Garcia-Pagan et al., 2009; Tandon et al., 2010). Randomized clinical trials show, however, that although NSBBs are effective in reducing portal pressure (PP) and the risk of bleeding from esophageal varices, around 15% of cirrhotic patients are intolerant of NSBB treatment, and up to 60% fail to achieve the treatment response required to prevent variceal bleeding defined as a fall in hepatic venous pressure gradient (HVPG) to less than 12 mmHg or a decrease of greater than 20% from baseline (Feu et al., 1995; Villanueva et al., 2001; Garcia-Pagan et al., 2009).

The renin angiotensin system (RAS) is an important mediator in the development of PHT (Bosch and Garcia-Pagan, 2000; Hennenberg et al., 2008; Lugo-Baruqui et al., 2010; Simoes et al., 2017). Increased angiotensin II (Ang II), the effector peptide of the classic RAS promotes intrahepatic vascular resistance, thus contributing to the pathogenesis of PHT (Schrier et al., 1988; Herath et al., 2007, 2009; Lubel et al., 2009). Although systemic administration of Ang II type 1 (AT1R) receptor blockers may improve PHT in early cirrhotic patients by reducing intrahepatic vascular resistance (Paizis et al., 2001; Kurikawa et al., 2003; Sookoian et al., 2005; Tandon and Garcia-Tsao, 2006; Tox and Steffen, 2006; Hennenberg et al., 2007; Kim et al., 2012), they have limited efficacy in advanced liver disease possibly due to the activation of other vasoconstrictive pathways such as the sympathetic nervous system (Tandon et al., 2010). Moreover, treatment with these drugs is associated with a number of off-target effects, including systemic hypotension and renal hypoperfusion (Heller et al., 2003, 2005; Schepke et al., 2008; Tandon et al., 2010).

**Figure 1:**
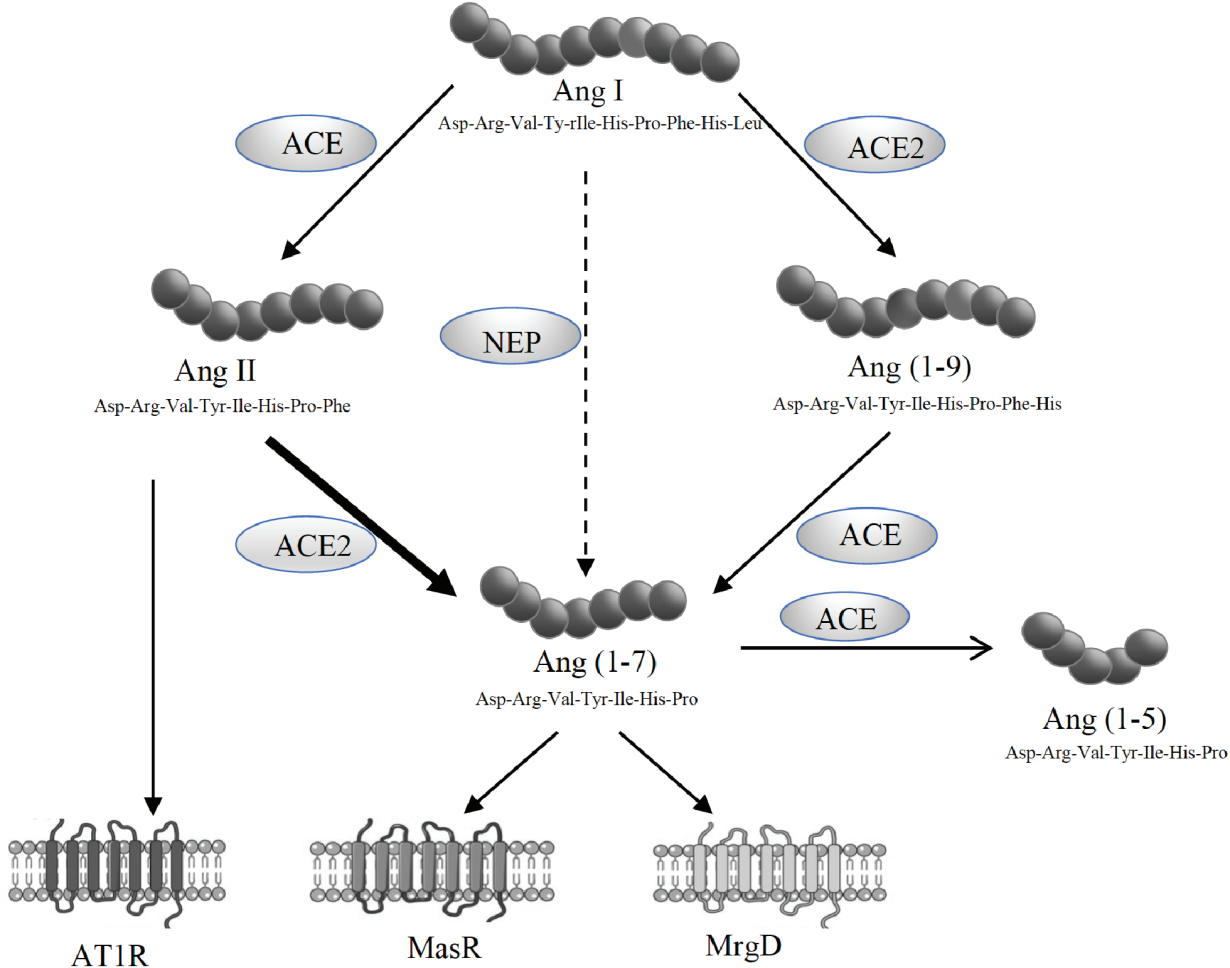
Schematic representation of the renin angiotensin system (RAS) showing the pathways responsible for the generation of angiotensin II (Ang II) and angiotensin-(1-7) [Ang-(1-7)]. Ang II acts *via* its type 1 receptor (AT1R) to exert vasoconstrictive effects. Ang II is degraded to Ang-(1-7) by angiotensin converting enzyme 2 (ACE2). Ang-(1-7) opposes Ang II effects through its receptors, MasR and MrgD.

The alternate axis of the RAS comprised of ACE2, angiotensin-(1-7) [Ang-(1-7)] and the Ang-(1-7) receptor Mas (MasR) (Figure 1). Ang-(1-7) has opposing effects to those produced by Ang II, including vasodilatory and anti-fibrotic properties, and has been shown to be protective in cardiovascular and renal tissues (Ferreira and Santos, 2005; Castelo-Branco et al., 2017; O’Neill et al., 2017). Although MasR has been shown to be the putative receptor for Ang-(1-7), it has also been proposed that MasR may interact with other angiotensin receptors such as AT1R and AT2R (Benter et al., 1993; Muthalif et al., 1998; Castro et al., 2005; Kostenis et al., 2005; Tesanovic et al., 2010; Castelo-Branco et al., 2017; O’Neill et al., 2017). However, we and others have recently reported that Ang-(1-7)-MasR axis is upregulated in the splanchnic circulation of cirrhotic animals and cirrhotic patients (Vilas-Boas et al., 2009; Grace et al., 2013). It has been shown that elevated Ang-(1-7) in the splanchnic vasculature contributes to splanchnic vasodilatation in experimental cirrhosis and the specific Mas receptor blocker A779 has been shown to increase splanchnic vascular resistance in cirrhotic rats (Grace et al., 2013). In patients, the potential role of the alternate RAS is supported by the finding that the Ang-(1-7)/Ang-II ratio in the splanchnic circulation is elevated and negatively correlated with systemic vascular resistance (Vilas-Boas et al., 2009). Recently, a further Ang-(1-7) receptor, the Mas-related G-protein coupled receptor type D (MrgD) has been identified (Tetzner et al., 2016), but its role in splanchnic vascular resistance has not been studied.

The goal of the current studies was to determine whether the MrgD may also play a role in splanchnic vasodilatation in portal hypertension. We also hypothesized that a combination treatment with Ang-(1-7) receptor blockers may counteract the systemic hypotensive effect of AT1R blockers in cirrhosis. We therefore studied the effect of Ang-(1-7) receptor blockade alone or in combination with AT1R blockade on portal pressure in animal models of cirrhotic and non-cirrhotic PHT.

## Experimental Procedure

### Chemicals and Drugs

Angiotensin (1-7), the Mas receptor blocker A779, and MrgD receptor blocker D-Pro^7^-Ang-(1-7) (D-Pro) were purchased from Mimotopes, Australia. The AT1R blocker losartan hydrochloride and angiotensin II type 2 receptor (AT2R) blocker PD123319 were purchased from Sigma-Aldrich, Australia. Components of a protease inhibitor mix (Grace et al., 2013), sodium-ethylenediaminetetraacetic acid (Na_2_EDTA), N-ethylmaleimide (NEM), aprotinin, leupeptin, and pepstatin were obtained from Sigma-Aldrich, Australia.

### Animal Models of Cirrhosis and Portal Hypertension

The experimental procedures in this study were approved by Austin Health Animal Ethics Committee and performed according to the National Health and Medical Research Council (NHMRC) of Australia guidelines for animal experimentation and the principles of the Helsinki declaration.

#### Bile Duct Ligation Model

Eight-week-old male Sprague Dawley rats (300–350 g BW) underwent bile duct ligation surgery (BDL) to induce cirrhosis and PHT. Rats were housed in a controlled environment with 12:12-h light to dark cycle with controlled temperature (22–24°C) and fed standard rat chow *ad libitum* (Norco, Lismore NSW, Australia) and water. After 1 week of acclimatization, the rats were anesthetized with isoflurane gaseous anesthesia (Therapon Pty Ltd., Victoria, Australia). The rats were also given a single dose of carprofen (5 mg/kg, Rimadyl, Australia) subcutaneously prior to surgery to limit post-operative discomfort. BDL surgery was performed as previously described (Herath et al., 2007). Briefly, a midline incision was made in the abdomen to enter the peritoneal cavity. The common bile duct was located and double ligated with 4/0 silk suture. The bile duct was then transacted between two ligatures. Intraoperatively, animals were kept warm on a heat pad and received 0.9% NaCl 10 ml/kg. The abdominal wall was closed in two layers using sterile 4/0 silk suture. Four weeks after BDL surgery, the rats were used for receptor blocker infusion experiments as described below.

#### Carbon Tetrachloride Model

Six-week-old male Sprague Dawley rats (200–250 g BW) were used to induce cirrhosis and PHT by injections of carbon tetrachloride (CCl_4_) as described previously (Fallowfield et al., 2014). Rats were housed in a controlled environment as described above. CCl_4_ was administered twice weekly *via* intraperitoneal injections, at a dose of 1 ml/kg, mixed with 1:1 ratio with olive oil. After 10 weeks of CCl_4_ administration, the rats were used for receptor blocker infusion experiments.

#### Partial Portal Vein Ligation Model

Eight-week-old male Sprague Dawley rats (300–350 g BW) were used to induce non-cirrhotic portal hypertension by partial portal vein ligation (PPVL) surgery as described previously (Kahn et al., 1988). Briefly, under gaseous anesthesia, a median laparotomy was performed and the portal vein was identified. A 19G needle was placed alongside the length of the portal vein, and a 4/0 silk suture was tied around the portal vein and the needle. The needle was then removed creating a calibrated stenosis in the portal vein, partially obstructing the portal flow. The abdominal wall was closed in two layers using sterile 4/0 silk suture. Two weeks after PPVL, the rats were used for receptor blocker infusion experiments.

### *In vivo* Pressure Measurement Experiments

Four weeks after BDL, 10 weeks after CCl_4_ and 2 weeks after PPVL, the rats were anesthetized with ketamine/xylazine mixture (75 and 10 mg/kg BW, respectively, Therapon Pty Ltd). A median laparotomy was performed, and a small PE 10 polyethylene catheter (Microtube Extrusions, NSW, Australia) was inserted into a small ileocecal vein and advanced to the portal vein to measure PP. A skin incision was made in the left inner thigh to identify the left femoral artery and cannulated with a PE 10 catheter to measure the mean arterial pressure (MAP). Portal vein and femoral artery catheters were connected to a highly sensitive pressure transducer (Zultek Engineering, Melbourne, VIC, Australia). The left femoral vein was cannulated with a similar PE 10 catheter for bolus injections of the receptor blockers. After insertion of the catheters, the rats were allowed to stabilize hemodynamically for 30 min prior to interventions.

**Figure 2:**
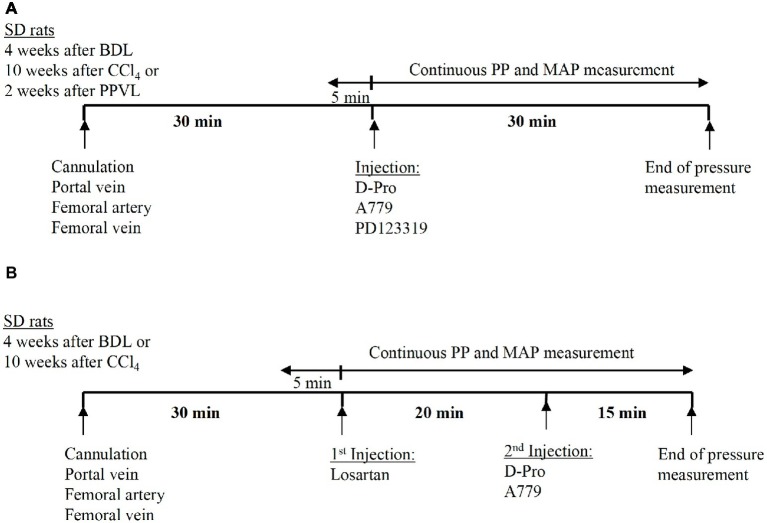
Schematic representation of the experimental plan adopted in the pressure measurement study. Thirty minutes after the cannulation of portal vein and femoral artery, the angiotensin receptor blockers were administered as a bolus injection *via* the femoral vein catheter. In the experiments using a single receptor blocker injection of either A779, D-Pro, or PD123319, pressure measurement was continued for 30 min after the injection **(A)**. In the experiments using combined treatment, losartan was injected first, followed by either A779 or D-Pro 20 min later. The pressure measurement was continued for a total of 35 min after losartan injection **(B)**.

The baseline values of PP and MAP were recorded prior to injection of the drugs. For a single receptor blocker experiments, either MasR blocker A779 (10 μg/kg) (Grace et al., 2013), MrgD blocker D-Pro (10 μg/kg), or AT2R blocker PD123319 (1 mg/kg) (Yang et al., 2011) was administered intravenously *via* the femoral vein catheter as a single bolus injection. The dose was calculated for each individual rat according to body weight. The bolus injection was prepared by dissolving a stock solution of 0.1 μg/kg (A779 or D-Pro) or 10 mg/ml (PD123319) in 0.9% NaCl to make a minimum injectable volume of 0.25 ml. This is to avoid variations in pressure due to adding volume to the circulation. After the bolus injection of the drug, the catheter was slowly flushed with 0.25 ml 20 IU/ml heparinized saline. Measurement of PP and MAP was continued for additional 30 min after injection (Figure 2A). Each treatment group of BDL, CCl_4_, and PPVL consisted of six to seven rats. For each model, a separate control group of animals (*n* = 6 per group) received saline injections for comparison with the treatment effects.

A separate set of experiments were carried out in the two cirrhotic PHT rat models (BDL and CCl_4_) to investigate whether the detrimental effects of AT1R blocker losartan on systemic arterial pressure can be counteracted by a second injection of either MasR or MrgD blockers; A779 or D-Pro, respectively. In this study, a bolus injection of losartan was administered and 20 min later, a bolus injection of either A779 or D-Pro was given. The pressure measurement was carried out for additional 20 min after the second injection (Figure 2B). A total of 18 BDL and 18 CCl_4_ rats were used (*n* = 6 per treatment group).

### Gene Expression Analysis of Mas Receptor, Mas-Related G-Protein Coupled Receptor Type D, and Angiotensin II Type-2 Receptor in Mesenteric Vascular Bed and Livers of Cirrhotic and Non-cirrhotic Rats

To determine the gene expression of RAS receptors, mesenteric arterial beds and livers were isolated from separate groups of rats at 4 weeks after BDL surgery, 10 weeks after CCl_4_ injections, 2 weeks after PPVL surgery, and age-matched control rats (*n* = 6–7 per each group). Total RNA was extracted using Trizol reagent (Sigma Aldrich, Sydney, Australia). Gene expression analysis of MasR, MrgD, and AT2R was carried out using quantitative real-time polymerase chain reaction (qPCR) as described previously (Herath et al., 2009; Grace et al., 2013).

### Immunohistochemical Staining of Mas Receptor and Mas-Related G-Protein Coupled Receptor Type D in the Liver of Cirrhotic and Non-cirrhotic Rats

Immunostaining for MasR and MrgD was performed in the sections (4 μm) obtained from 4% paraformaldehyde (PFA) fixed paraffin embedded liver tissues of the CCl_4_, PPVL, and age-matched control rats (Melbourne histology platform, The University of Melbourne). Primary monoclonal antibodies for MasR (Alomone labs, Israel) and MrgD (Alomone labs, Israel) were used at 1:500 and 1:400 dilutions, respectively. Secondary goat anti-mouse antibody (Sigma-Aldrich) was used at a dilution of 1:500. Positive signals were detected by incubation of these sections with DAB chromogen for 10 min at room temperature. Sections were counterstained with hematoxylin for 15 s and mounted on glass slides to visualize under microspore at X20 magnifications.

### *In vivo* Angiotensin (1-7) Peptide Metabolism Assay

To determine the longevity of the angiotensin peptides in the rat circulation, a separate set of experiments was carried out using BDL rats. Four weeks after BDL, the rats were anesthetized with ketamine/xylazine mixture and the femoral artery and vein were cannulated using PE-10 catheters as described previously (Grace et al., 2013). After 30 min of stabilization, 1 ml of blood was drawn from the femoral artery as the baseline sample into an Eppendorf tube containing protease inhibitor mix (20 μl/ml of blood: 50 mmol/L Na2 EDTA, 0.2 mol/L N-ethylmaleimide, 21,000 U/ml aprotonin, 5 mg/ml leupeptin, and 1 mg/ml pepstatin; prepared fresh daily and kept on ice) to prevent breakdown of angiotensin peptides. Injection of either MasR or MrgD blockers, A779 or D-Pro (10 μg/kg), respectively, was performed thereafter *via* the femoral vein catheter. Blood samples were collected at 30 s, 1, 2, and 5 min after injection. A separate group of rats was injected with Ang-(1-7) peptide (10 μg/kg) and blood was collected as before. After completion of the experiment, the rats were euthanized by exsanguination. Blood samples were centrifuged at 4,000 rpm for 10 min to harvest plasma. Radioimmunoassay for Ang-(1-7) (Prosearch International, Australia, Pty Ltd) was performed to determine plasma concentration of peptide blockers and Ang-(1-7) at different time points. Twelve BDL rats were used for the experiment, with four rats in each of the three treatment groups. The extent of cross-reactivity for D-Ala (A779) and D-Pro was not determined for the Ang-(1-7) radioimmunoassay.

### Statistical Analysis

Data were analyzed using paired *t*-test and repeated-measures ANOVA with Tukey *post hoc* test where appropriate. Results are expressed as mean ± SEM. Statistical analyses were performed using GraphPad Prism 7.0. Values less than 0.05 were considered as statistically significant.

## Results

### Mas Receptor and Mas-Related G-Protein Coupled Receptor Type D Antagonism Reduce Portal Pressure in Cirrhotic Rats

**Figure 3:**
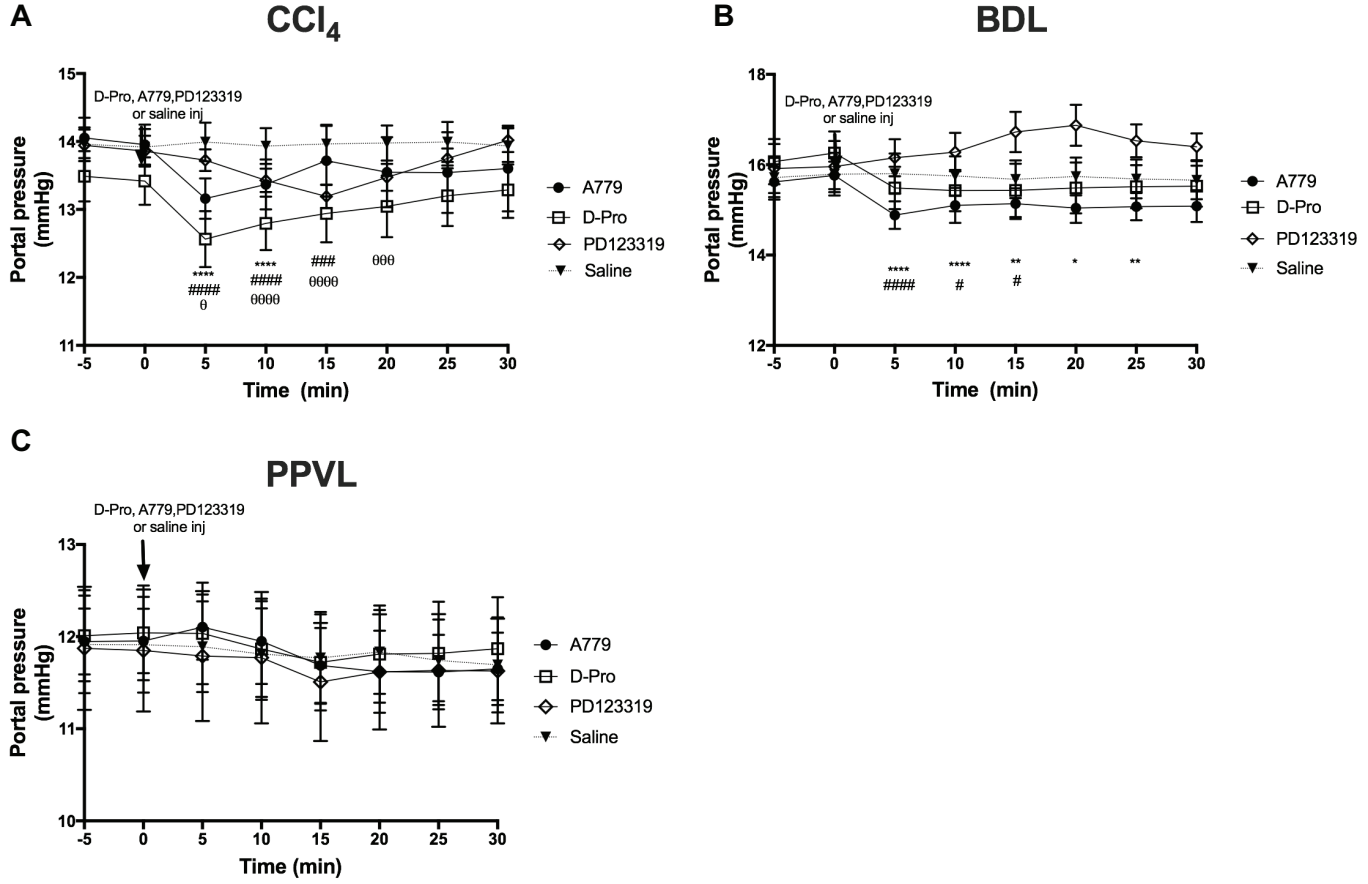
Changes in portal pressure (PP) after intravenous bolus injections of either the MasR blocker A779 (10 μg/kg), MrgD blocker D-Pro (10 μg/kg), or AT2R blocker PD123319 (1 mg/kg) in CCl_4_ **(A)**, BDL **(B)**, and PPVL **(C)** models. Saline injection served as the control. Pressure measurement was commenced 5 min prior to injection and continued for 30 min after the injection. Each time point represents the mean ± SEM profile from six to seven rats per treatment group. ^****^p < 0.05, ^**^p < 0.01, ^*^p < 0.05 baseline vs. A779, ^####^*p* < 0.05, ^###^*p* < 0.005, ^#^*p* < 0.05 baseline vs. D-Pro, ^θθθθ^*p* < 0.05, ^θθθ^*p* < 0.01, ^θ^*p* < 0.05 baseline vs. PD123319.

A bolus injection of both MasR blocker A779 and MrgD blocker D-Pro significantly (*p* < 0.001) reduced PP when measured at 5 min post-injection in both CCl_4_ (Figure 3A) and BDL (Figure 3B) rats. In BDL model, the maximum reduction in portal pressure was 5.8% for A779 and 4.8% for D-pro, while in CCl_4_ model, it was 5.7 and 6.4% for A779 and D-pro, respectively. The reduction in PP persisted for up to 15 min after the injection of D-Pro in both BDL and CCl_4_ models, while after the injection of A779, it persisted for 10 min in the CCl_4_ and 25 min in BDL models. In the CCl_4_ model, AT2R blockade with PD123319 also resulted in a significant reduction in PP at 5 min, which lasted for up to 20 min after the bolus injection. The maximum reduction in pressure was 4.8% in CCl_4_ model. In contrast, A779, D-Pro, or PD123319 failed to reduce PP in the non-cirrhotic PPVL rat model (Figure 3C).

### Mas Receptor and Mas-Related G-Protein Coupled Receptor Type D Antagonism Increased Mean Arterial Pressure in Cirrhotic Bile Duct Ligated Rats

**Figure 4:**
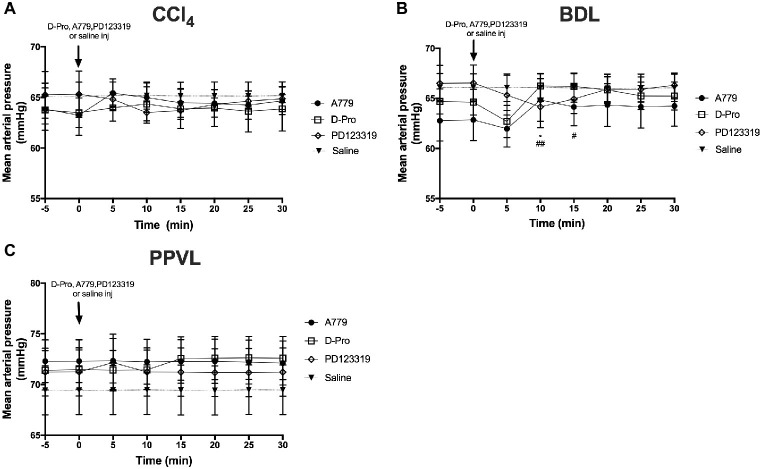
Changes in mean arterial pressure (MAP) with intravenous bolus injections of either MasR blocker A779 (10 μg/kg), MrgD blocker D-Pro (10 μg/kg) or AT2R blocker PD123319 (1 mg/kg) in CCl_4_ **(A)** BDL **(B)**, and PPVL **(C)** models. Saline injection served as the control. Each time point represents the mean ± SEM profile from six to seven rats per treatment group. ^*^p < 0.05 baseline vs. A779; ^##^*p* < 0.01, ^#^*p* < 0.05, baseline vs. D-Pro.

There was no effect of the blockers on MAP in the CCl_4_ model (Figure 4A). However, in the BDL model, there was an overall effect with time (*p* < 0.05) which was dependent (*p* < 0.01) on the treatment groups (Figure 4B). Thus, in comparison with baseline pressure at time 0, both A779 and D-Pro caused a small (1–2 mmHg) but significant (*p* < 0.05) increase in MAP (Figure 4B) at 10 min after the injection, which persisted for up to 15 min in D-Pro injected animals. The blockers had no effect on MAP in PPVL rats (Figure 4C). Moreover, the AT2R blocker PD123319 had no effect on MAP in any of the models investigated.

### Mas Receptor and Mas-Related G-Protein Coupled Receptor Type D Antagonism Fail to Prevent Angiotensin II Type 1 Receptor Blockade-Induced Reduction in Mean Arterial Pressure in Cirrhotic Rats

**Figure 5:**
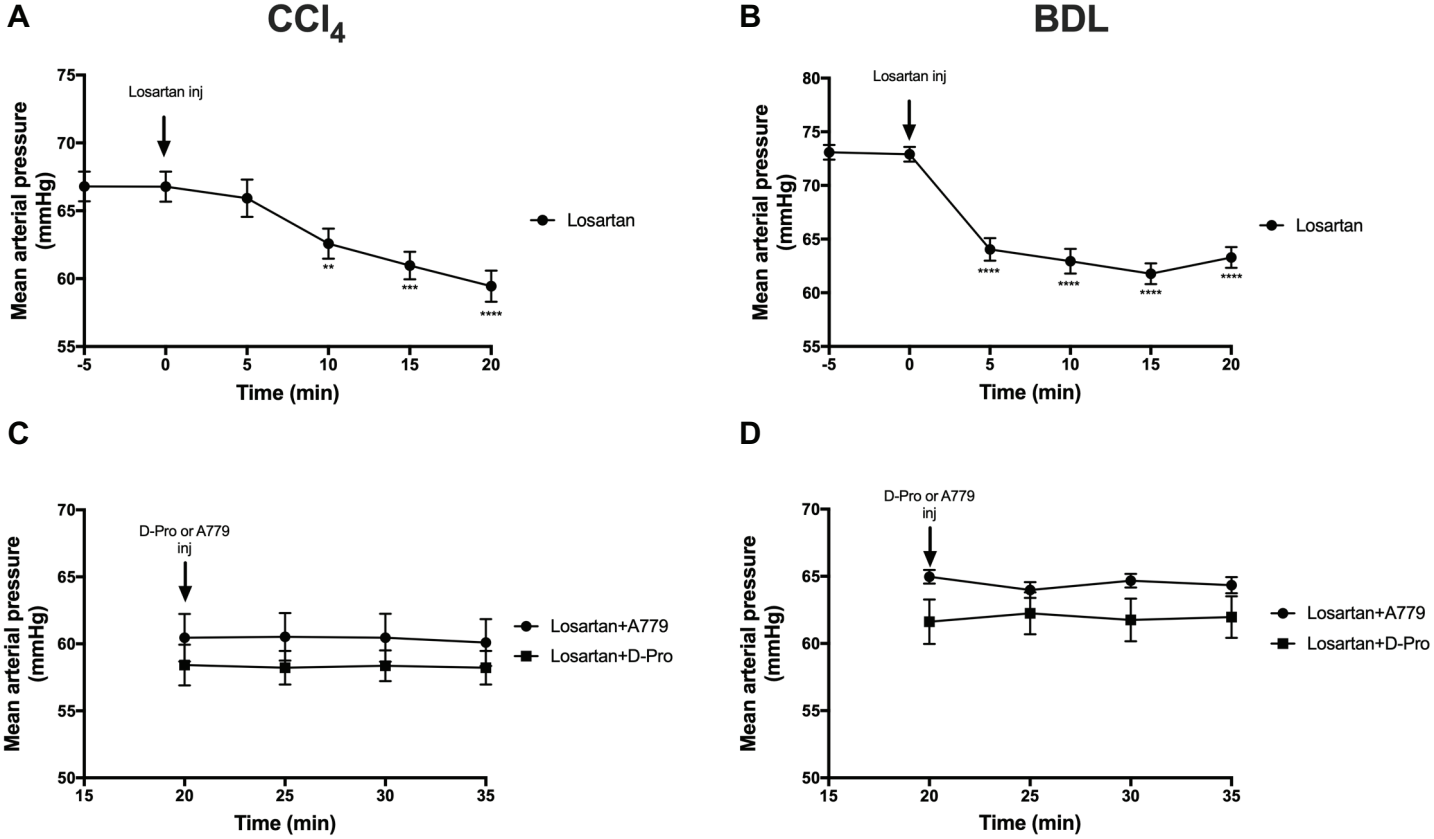
Changes in mean arterial pressure after the treatment with AT1R blocker losartan (1 mg/kg) in the CCl_4_ **(A)** and BDL **(B)** models. Note that the two groups that received losartan injections in each model were pooled for t-test analysis. Bottom panels show mean arterial pressure of the two losartan groups after receiving either MasR blocker A779 (10 μg/kg) or MrgD blocker D-Pro (10 μg/kg) in the CCl_4_ **(C)** and BDL **(D)** models. Losartan significantly reduced MAP in all groups; however, A779 or D-Pro which was given 20 min after losartan failed to counteract the hypotensive effect of losartan. Each time point represents the mean ± SEM profile from 12 to 14 **(A,B)** or 6 to 7 **(C,D)** rats per treatment group. Data in **(C)** and **(D)** were analyzed by repeated-measures ANOVA. ^**^p < 0.01, ^***^p < 0.001, ^****^p < 0.0005 baseline vs. post-losartan injection.

Administration of the AT1R blocker losartan significantly (*p* < 0.01) reduced MAP in the CCl_4_ model starting at 10 min post-injection (Figure 5A) and in the BDL model starting at 5 min post-injection (Figure 5B). This marked reduction in MAP from baseline pressure remained significantly low thereafter. However, a bolus injection of A779 or D-Pro given 20 min after losartan injection failed to significantly increase MAP in the CCl_4_ (Figure 5C) and BDL (Figure 5D) models.

### Angiotensin II Type 1 Receptor Antagonism With Losartan Reduces Portal Pressure

**Figure 6:**
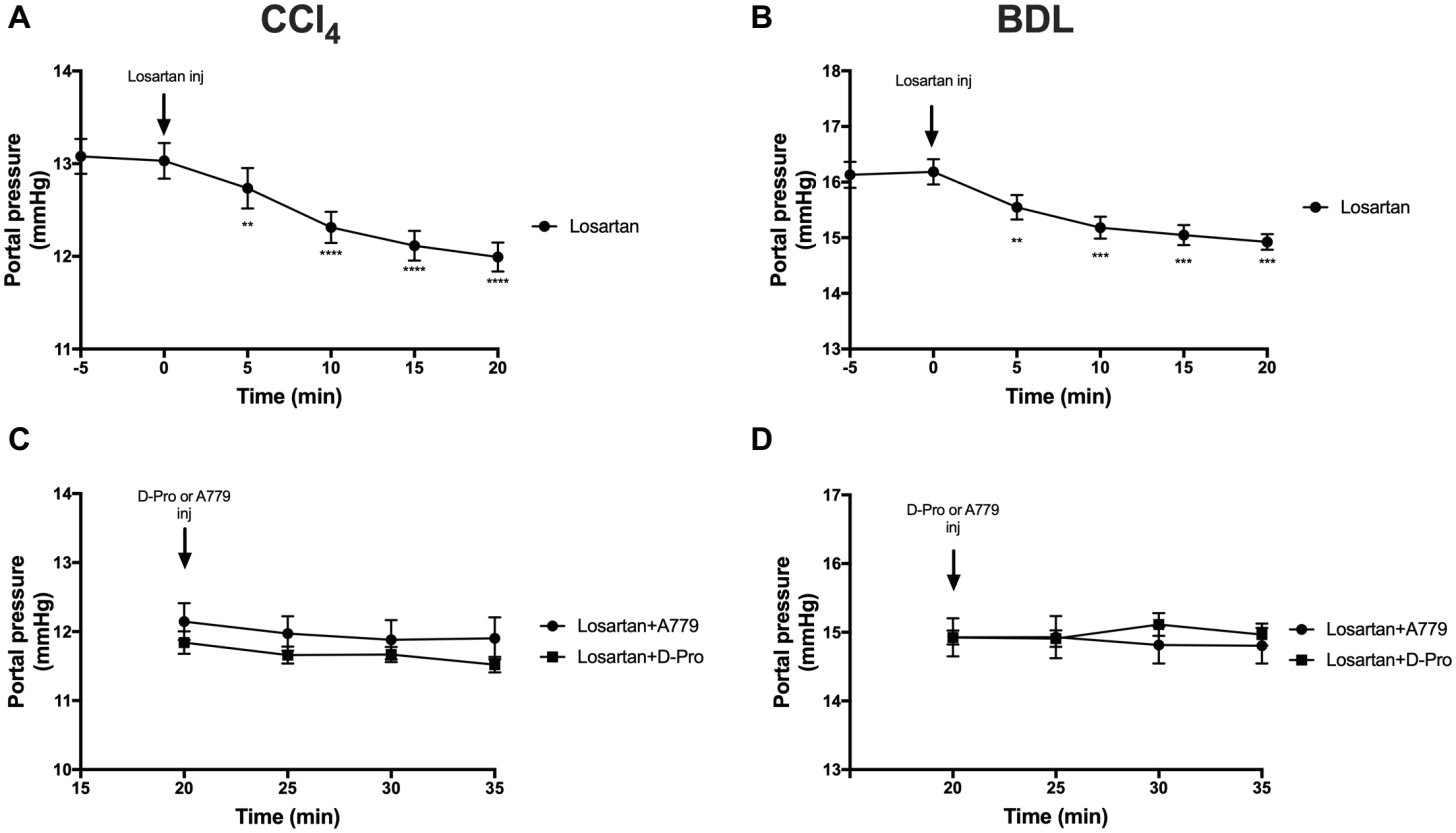
Changes in portal pressure after the treatment with AT1R blocker losartan (1 mg/kg) in the CCl_4_ **(A)** and BDL **(B)** models. Note that the two groups that received losartan injections in each model were pooled for *t*-test analysis. Bottom panels show portal pressure of the two losartan groups after receiving either MasR blocker A779 (10 μg/kg) or MrgD blocker D-Pro (10 μg/kg) in the CCl_4_ **(C)** and BDL **(D)** models. Losartan significantly reduced portal pressure in all groups; however, A779 or D-Pro which was given 20 min after losartan failed to affect portal pressure. Each time point represents the mean ± SEM profile from 12 to 14 **(A,B)** or 6 to 7 **(C,D)** rats per treatment group. Data in **(C)** and **(D)** were analyzed by repeated-measures ANOVA. ^**^p < 0.01, ^***^p < 0.001, ^****^p < 0.0005 baseline vs. post-losartan injection.

Administration of the AT1R blocker losartan produced a significant (*p* < 0.01) reduction in PP from baseline pressure in the CCl_4_ (Figure 6A) and BDL (Figure 6B) models starting at 5 min post-injection and remained significantly low thereafter. However, a bolus injection of A779 or D-Pro given 20 min after losartan injection failed to significantly reduce PP in the CCl_4_ (Figure 6C) and BDL (Figure 6D) models.

### *In vivo* Angiotensin Peptide Metabolism

Unlike AT1R blockers which are non-peptide compounds, MasR and MrgD blockers are peptide derivatives and are very similar in structure to Ang-(1-7) where MasR blocker A779 has D-alanine at position 7 and MrgD blocker D-Pro-Ang-(1-7) has D-proline instead of alanine at position 7. They are therefore expected to have a very short half-life in blood circulation as they are subject to breakdown by proteases (Yamada et al., 1998; Trask and Ferrario, 2007). Therefore, a single bolus injection of these drugs may not be adequate to have a long-lasting effect on PP in these rats. We therefore investigated plasma concentrations of these blocking peptides in the rat circulation at different time points after intravenous bolus injections.

**Figure 7:**
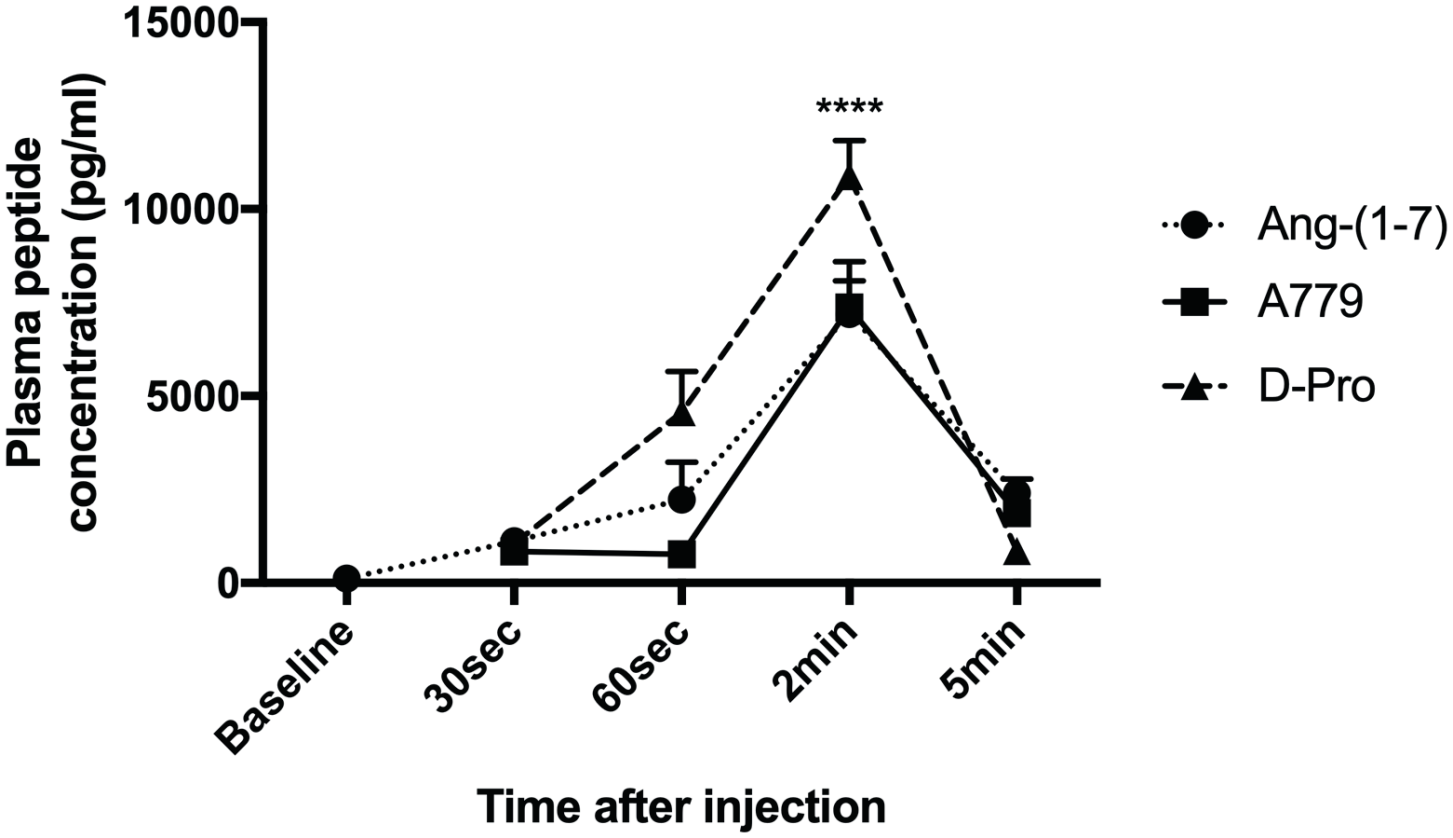
Plasma concentrations of Ang-(1-7) peptide (10 μg/kg) and peptide receptor blockers A779 (10 μg/kg) and D-Pro (10 μg/kg) after a bolus injection of intravenous administration. Each time point represents the mean ± SEM profile from 4 rats per treatment group. ^****^p < 0.005 concentration of each peptide at 2 min vs. baseline.

Mean baseline concentrations of Ang (1-7) in the three rat groups before receiving a bolus injection of either Ang-(1-7), A779, or D-pro were 119.73, 91.98, and 134.33 (pg/ml), respectively. Following an intravenous injection of Ang-(1-7), A779, or D-Pro, plasma concentrations of the respective peptides peaked 2-min post-injection (Figure 7). However, the increased circulating peptide levels were then started to decline and returned to baseline levels within 5 min after injection, suggesting that they have a very short-term activity in the rat circulation. However, the effects invoked by these blockers appeared to last for up to 25 min before returning to baseline, suggesting that greater effects may be seen after a continuous infusion of the blockers.

### Mas Receptor and Mas-Related G-Protein Coupled Receptor Type D Gene Expressions Are Upregulated in Cirrhotic Mesenteric Vessels

**Figure 8:**
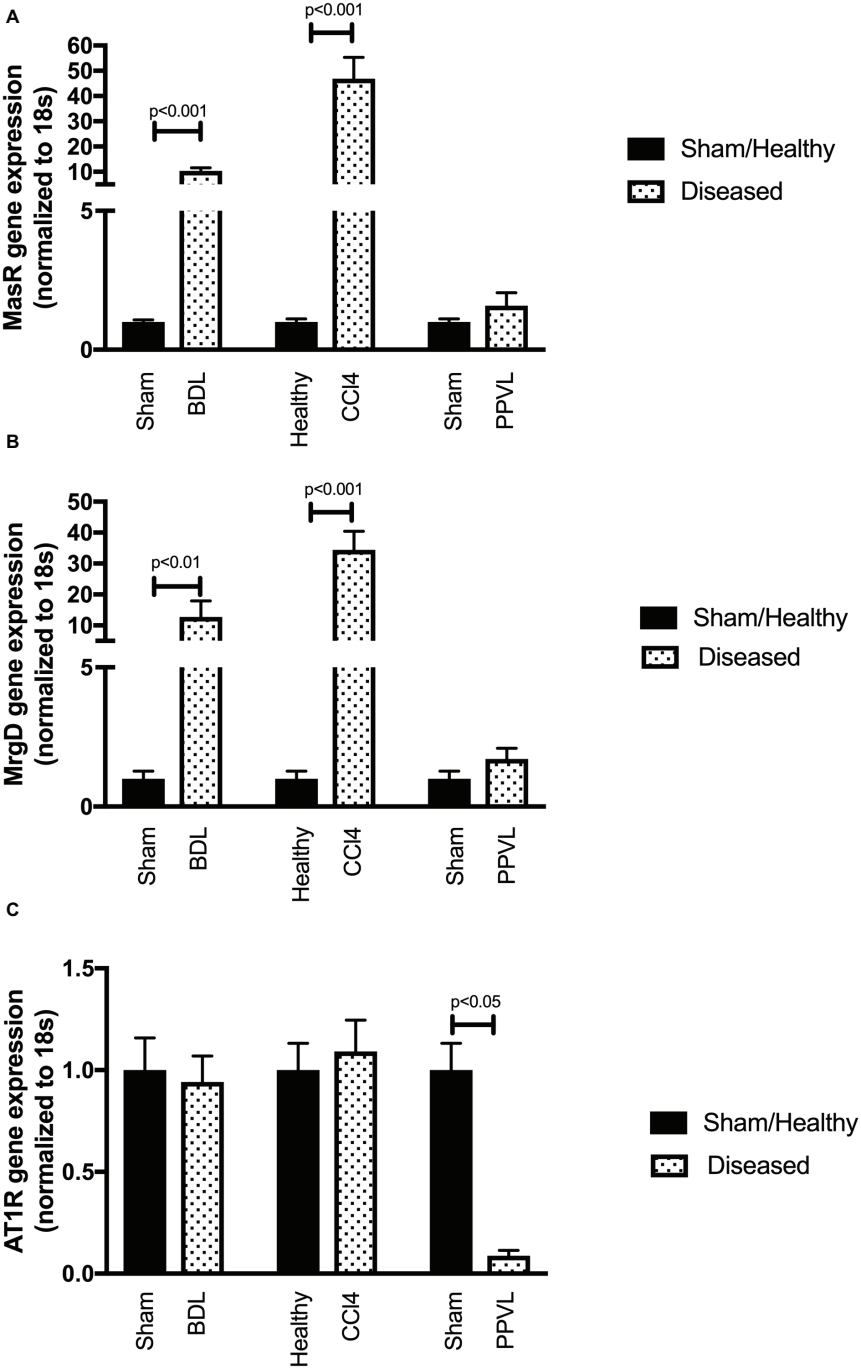
Receptor gene expression of MasR **(A)**, MrgD **(B)**, and AT1R **(C)** analyzed by qPCR in mesenteric vascular bed of the CCl_4_, BDL and PPVL rats compared with sham-operated and healthy control rats. Data have been normalized to endogenous control gene 18S, and healthy control group was given an arbitrary value of 1. Each time point represents the mean ± SEM profile from six to seven rats per treatment group.

Gene expression of MasR (Figure 8A) and MrgD (Figure 8B) were upregulated in the mesenteric vascular bed of both CCl_4_ and BDL models, suggesting that both receptors are likely to regulate Ang-(1-7) mediated splanchnic vasodilatation in cirrhosis. In contrast, AT2R expression was at undetectable levels (data not shown) in mesenteric vessels, which suggest that AT2R is unlikely to play a role in splanchnic vasodilatation. Despite this, the assumed AT2R-specific blocker, PD123319, was effective in reducing splanchnic vasodilatation, as reflected by a reduction in PP (see Figure 3). This raises a concern about the specificity of PD123319 and appears that it might also bind to other receptors, in particular, MasR and/or MrgD (Tetzner et al., 2016). On the other hand, the expression of MasR and MrgD was not changed in the mesenteric vessels of non-cirrhotic PPVL rats (Figures 8A,B), supporting the argument that the alternate RAS is not a key mediator in this condition. However, in PPVL rats, mesenteric vasodilatation likely results in with the downregulation of the classical RAS, as reflected by the downregulated AT1R expression in PPVL mesenteric vessels (Figure 8C). Conversely, AT1R expression is not altered in the mesenteric vessels of cirrhotic BDL and CCl_4_ rats, suggesting AT1R may not play a role in regulating the resistance within cirrhotic mesenteric vessels.

### Mas Receptor but Not Mas-Related G-Protein Coupled Receptor Type D Gene Expression Is Upregulated in the Cirrhotic Livers

**Figure 9:**
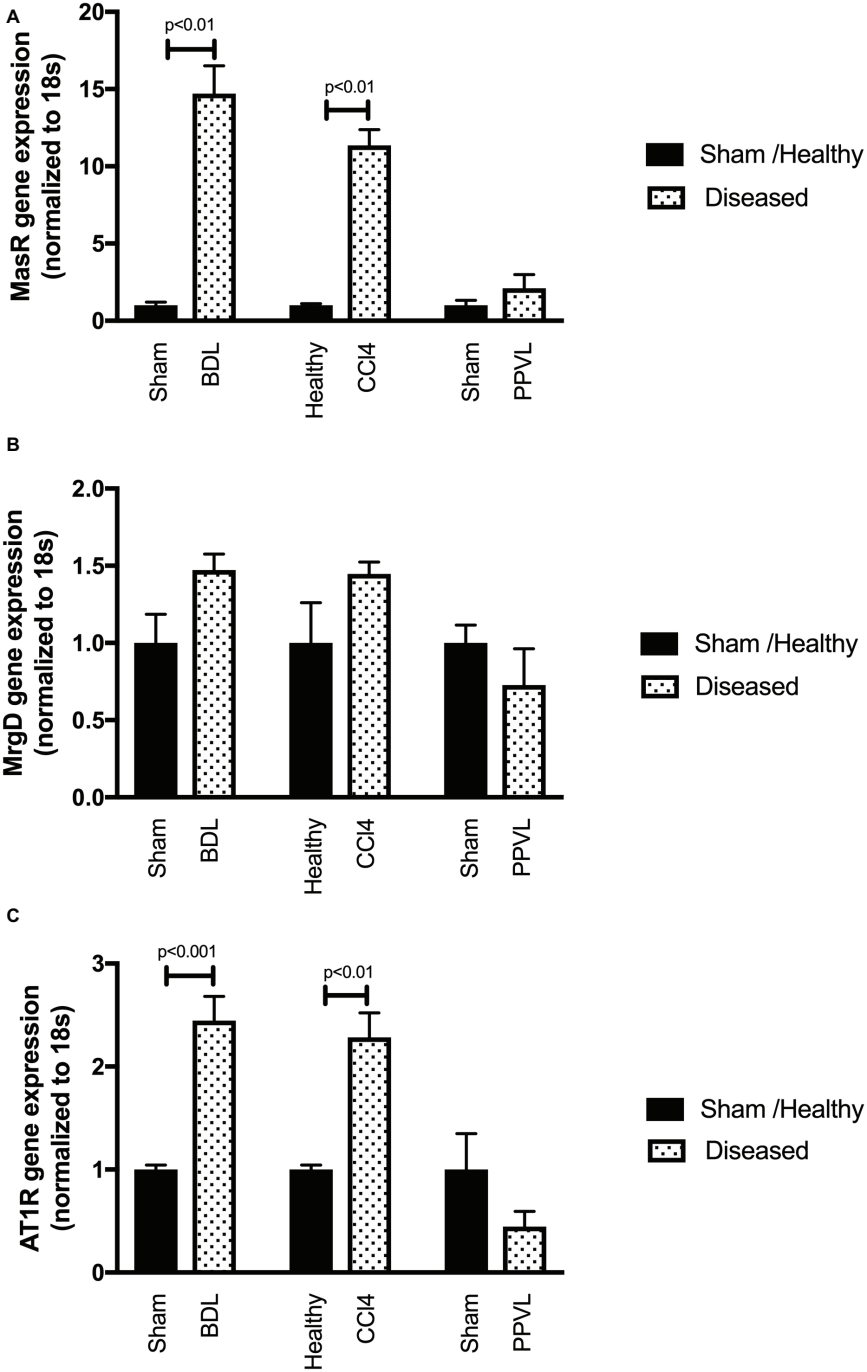
Receptor gene expression of MasR **(A)**, MrgD **(B)**, and AT1R **(C)** analyzed by qPCR in the livers of the CCl_4_, BDL, and PPVL rats compared with sham-operated and healthy control rats. Data have been normalized to endogenous control gene 18S, and healthy control group was given an arbitrary value of 1. Each time point represents the mean ± SEM profile from six to seven rats per treatment group.

Gene expression of MasR (Figure 9A) was upregulated in the cirrhotic livers of both CCl_4_ and BDL rats compared to healthy controls. However, in contrast, MrgD (Figure 9B) expression was not changed in the livers of both CCl_4_ and BDL rats. These findings suggest that although MasR likely contributes to the regulation of hepatic vascular resistance in cirrhosis, MrgD, on the other hand, unlikely to play a role in regulating the resistance within the cirrhotic livers. The AT1R gene expression was also upregulated in the cirrhotic livers of both CCl_4_ and BDL rats, suggesting the prominent role of AT1R in regulating the resistance within the cirrhotic livers. However, similar to that in the mesenteric vascular bed, AT2R was not detectable (data not shown) in these livers, suggesting that AT2R has a minimal activity within the hepatic vascular bed as well. On the other hand, the expression of neither MasR, MrgD, nor AT1R was changed in non-cirrhotic livers of the PPVL rats (Figures 9A–C).

### Mas Receptor but Not Mas-Related G-Protein Coupled Receptor Type D Protein Localized to Liver Sinusoids of Cirrhotic Rats

**Figure 10:**
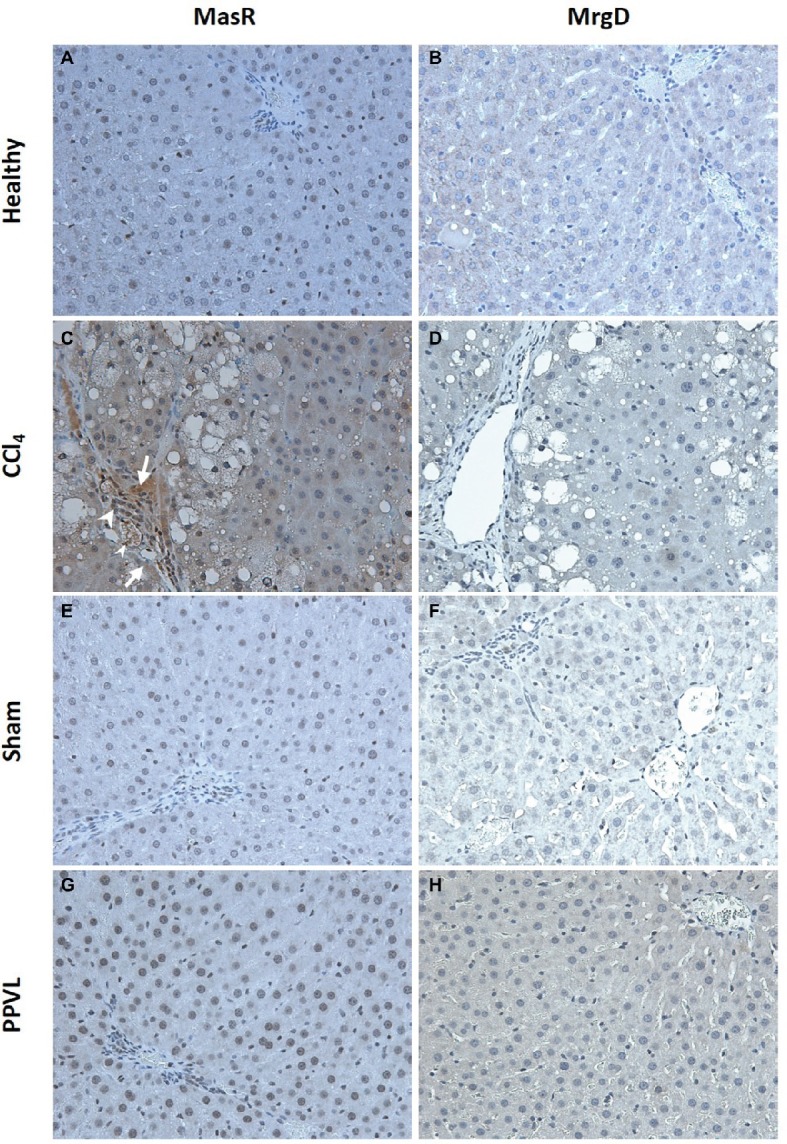
Immunohistochemical localization of MasR (left column) and MrgD (right column) in the liver. MasR **(C)** and MrgD **(D)** staining of CCl_4_ livers were compared with those of healthy control livers (**A,B**, respectively). Note that in cirrhotic livers **(C)**, there was strong positive staining for MasR in liver sinusoids (arrow) which is consistent with the localization of hepatic stellate cells and/or liver sinusoidal endothelial cells. Also note that positive staining for MasR in bile duct epithelial cells (arrow head-large) and hepatic arterioles (arrow head-small) in the cirrhotic liver. However, there was no positive staining for MrgD in the cirrhotic liver **(D)**. Livers from PPVL rats showed no positive staining for both receptors **(G,H)** compared to the controls **(E,F)**. Original images were captured at the X20 magnification.

Cirrhotic livers of CCl_4_ rats show prominent staining of MasR when compared to the healthy livers obtained from the olive oil injected rats (Figures 10A,C). Positive MasR staining in cirrhotic livers was prominent in liver sinusoids, consistent with the localization of sinusoidal endothelial cells and/or hepatic stellate cells (Figure 10C). In addition, there was strong staining of MasR in bile duct epithelial cells and hepatic arterioles (Figure 10C). However, in a marked contrast, MrgD staining in cirrhotic CCl_4_ livers showed no difference to that of the healthy control livers (Figures 10B,D). Protein expression of MasR and MrgD supports the gene expression data, where MasR but not MrgD was upregulated in the cirrhotic liver (see Figure 9), confirming that MasR but not MrgD has a prominent role in regulating hepatic vascular resistance in cirrhosis. On the other hand, neither MasR nor MrgD protein expression was changed in non-cirrhotic livers of PPVL rats (Figures 10G,H) compared to that of sham operated controls (Figures 10E,F).

## Discussion

The present study demonstrates that blockade of the receptors of the alternate RAS is effective in reducing portal pressure in cirrhosis. Peptide-derived blockers such as A779 and D-Pro reduce portal pressure *via* inhibition of vasodilatory MasR and MrgD, which is likely associated with a subsequent increase in splanchnic vascular resistance, resulting in reduced portal blood flow (Grace et al., 2013) However, a bolus dose of the blockers was unable to produce a clinically significant reduction (>20% from baseline) in portal pressure, which otherwise suggests that continuous infusions of the blockers may be effective in producing a clinically significant effect on portal pressure in these models. Nevertheless, the present findings suggest that the MasR and MrgD are potential targets for the development of therapies to treat cirrhotic portal hypertension. Moreover, the findings demonstrate that the blockers of the alternate RAS are ineffective in non-cirrhotic portal hypertension, suggesting that mechanisms other than the alternate RAS activation may contribute to pathological splanchnic vasodilatation in this condition.

The present study aimed at manipulating the RAS in portal hypertension with specific focus on the alternate axis of this system. The effects of Ang-(1-7), the effector peptide of the alternate RAS, are mediated through its putative receptor Mas (Santos et al., 2003). In the present study, blockade of MasR, whose expression in cirrhotic mesenteric vessels was upregulated, reduced portal pressure in cirrhotic rats. This supported our published work that MasR blockade increased splanchnic vascular resistance in cirrhotic rats, leading to a reduced portal pressure and subsequent improvement in portal hypertension (Grace et al., 2013). Although the MasR was initially considered to be a specific receptor for Ang-(1-7) (Santos et al., 2003), several studies have proposed the existence of an alternate receptor for this peptide by demonstrating that the effects of Ang-(1-7) cannot be completely abolished by the specific MasR blocker A779 (Silva et al., 2007; Gembardt et al., 2008; Grace et al., 2013; Lautner et al., 2013). Indeed, an Ang-(1-7)-induced reduction in hepatic perfusion pressure in the *in-situ* perfused cirrhotic rat liver was blocked by D-Pro but not by A779, suggesting that there was a receptor population that was sensitive to D-Pro (Herath et al., 2009). Support for these findings comes from a recent study which reported the identification of MrgD as an alternate receptor for Ang-(1-7) (Lautner et al., 2013). This was followed by a study which showed that in addition to MasR, vasodilatory effects of Ang-(1-7) can also be mediated through MrgD (Tetzner et al., 2016).

Although the role of MrgD in splanchnic vasodilatation in cirrhosis was largely unknown, we hypothesized that this receptor may share similar properties to its counterpart MasR and that MrgD blockade would also be expected to improve portal hypertension in cirrhotic rats. We now report for the first time that MrgD, a recently characterized vasodilatory receptor of the alternate RAS, is markedly upregulated in cirrhotic mesenteric vessels. It appears to play a role in pathological vasodilatation in cirrhotic portal hypertension since MrgD blockade with D-Pro significantly reduced portal pressure in both CCl_4_ and BDL cirrhotic rat models. Moreover, the importance of MrgD as a mesenteric vasculature-specific receptor is highlighted by the fact that MrgD gene and protein expression in the cirrhotic liver was not changed, suggesting that MrgD blockade likely have a minimal role on hepatic vascular resistance in cirrhosis. However, in marked contrast, MasR gene expression was upregulated in the cirrhotic livers. Moreover, positive MasR staining in cirrhotic livers was localized to liver sinusoids, which is consistent with the location of hepatic stellate cells and/or sinusoidal endothelial cells. This confirms our previous reports (Grace et al., 2013) and may suggest that MasR blockade may negatively affect portal pressure by increasing hepatic vascular resistance (Grace et al., 2013). However, in the present study, we found that MasR blockade reduced portal pressure in cirrhotic rats, probably reflecting that its negative effect within the hepatic vasculature might have outweighed by its positive and beneficial effects on the splanchnic vascular bed in our current experimental settings.

The role of Ang-(1-7)/MasR axis in mediating systemic vascular resistance has been previously demonstrated (Sampaio et al., 2003; Santos et al., 2003, 2006; Castro et al., 2006; Botelho-Santos et al., 2007; Pinheiro et al., 2009). It was reported that while Ang-(1-7) infusion in rats reduces peripheral vascular resistance (Sampaio et al., 2003; Botelho-Santos et al., 2007), MasR deficiency in mice increased the resistance in coronary (Castro et al., 2006; Santos et al., 2006) and renal vasculature (Santos et al., 2003; Pinheiro et al., 2009). We therefore also sought to determine and compare the effects of MasR and MrgD blockers on systemic vascular resistance by measuring MAP. We found that A779 and D-Pro administration caused a small but significant increase in MAP in BDL rats, suggesting that both MasR and MrgD blockers likely increase systemic vascular resistance in cirrhosis. This agrees with our previous findings that MasR blockade not only increased splanchnic vascular resistance but also increased hepatic and renal vascular resistance without affecting the cardiac output in cirrhotic rat models (Grace et al., 2013).

Although A779 and D-Pro are effective in reducing portal pressure, similar to Ang-(1-7), A779 and D-Pro are also peptide derivatives, and therefore, it is possible that they have a very short half-life in the circulation. Although the pharmacokinetics of Ang-(1-7) was extensively studied (Yamada et al., 1998; Trask and Ferrario, 2007), the fate of circulating A779 or D-Pro has not been studied to date. This has prompted us to measure circulating concentrations of the blockers following a bolus injection and found that A779 and D-Pro are rapidly degraded in the rat circulation, possibly by the activity of protease enzymes. Despite their rapid metabolism in the circulation, the portal pressure lowering effects were however sustained for up to 25 min before returning to baseline level, suggesting that greater effects may be seen after a continuous infusion of the blockers.

Development of non-cirrhotic portal hypertension has been closely linked to excessive splanchnic vasodilatation (Chojkier and Groszmann, 1981; Vorobioff et al., 1983); however, the contribution of the RAS in this condition is unknown. It has been shown that splanchnic vasodilatation in non-cirrhotic portal hypertension is also mediated through enhanced activity of nitric oxide in the mesenteric vascular bed (Pizcueta et al., 1992; Hori et al., 1998; Tsai et al., 2003). Because Ang-(1-7) acts *via* the MasR and/or MrgD to activate a cascade of downstream signaling pathways to release vasodilatory molecules including nitric oxide (Dimmeler et al., 1999; Mount et al., 2007; Grace et al., 2013), we hypothesized that similar to cirrhotic portal hypertension, the alternate RAS contributes to splanchnic vasodilatation in non-cirrhotic portal hypertension. Contrary to this, we found that in the PPVL rat model of non-cirrhotic portal hypertension, blockade of neither MasR nor MrgD, whose mesenteric expression was unaltered, affected portal pressure or MAP, suggesting that unlike in cirrhotic portal hypertension, the alternate RAS is not a key mediator of splanchnic vasodilatation in this condition. On the other hand, we found that AT1R, which is the receptor for vasoconstrictive peptide Ang II, is significantly downregulated in the mesenteric vessels of the PPVL rats, suggesting classical but not alternate RAS appears to be involved in the pathogenesis of portal hypertension in this model.

Although it is well known that Ang (1-7) produces its effects by binding to MasR and MrgD (Santos et al., 2003; Tetzner et al., 2016), early cell culture studies have reported some actions of Ang-(1-7) were mediated *via* the subtypes of AT1R (Muthalif et al., 1998; Heitsch et al., 2001). Some studies also proposed that MasR may interact with AT1R by receptor hetero-dimerization or alterations in post-receptor signaling (Castro et al., 2005; Kostenis et al., 2005; Tesanovic et al., 2010; Castelo-Branco et al., 2017; O’Neill et al., 2017), which in turn leads to the inhibition of Ang II activity while promoting those of Ang-(1-7). Conversely, many studies have shown that Ang-(1-7) has a low binding affinity to AT1R compared to Ang II (Rowe et al., 1995; Tallant et al., 1997). Moreover, several *in vivo* and *in vitro* studies have documented the specificity of these drugs at the doses employed in the current study such that, AT1R blocker losartan is not a ligand for MasR (Santos et al., 2003) and A779 also has no affinity to AT1R (Fontes et al., 1994; Santos et al., 1994; Bosnyak et al., 2011).

There is some evidence that ATR2 also mediates Ang-(1-7) action. This was based on observations that the vasodilatory effects of Ang-(1-7) *in vivo* (Heitsch et al., 2001) and *ex vivo* and *in vitro* (Tesanovic et al., 2010; Grace et al., 2013) were inhibited by AT2R blockade with PD123319. We report here that similar to that of the MasR and MrgD blockers, a bolus injection of AT2R blocker PD123319 also reduced portal pressure in the cirrhotic CCl_4_ model, despite undetectable AT2R expression in cirrhotic mesenteric vessels. This suggests that AT2R is unlikely to be responsible for portal pressure lowering effect of PD123319. This is supported by recent studies which showed that Ang-(1-7)-stimulated cyclic adenosine monophosphate (cAMP) release by HEK293 cells transfected with MasR or MrgD was completed blocked by PD123319 (Tetzner et al., 2016), pointing to a possibility that MasR and/or MrgD can also be targets for PD123319. In line with this, AT2R blocker PD123319 also reduced portal pressure in both cirrhotic rat models, but pressure reduction in BDL rats was not statistically significant. This difference could be explained by the differences in gene expression in mesenteric vessels. The expression of MrgD or MasR in mesenteric vessels of CCl_4_ rats was approximately 40-fold increased, whereas it was only about 10- to 15-fold in BDL vessels, pointing to the possibility that the non-specific effect of PD123319 may be stronger on CCl_4_ vessels than in BDL vessels. On the other hand, both these receptors were not upregulated in the mesenteric vessels of PPVL rats, which clearly explain the absence of drug effects in this model.

Therapies targeting hepatic fibrosis and/or intrahepatic vascular resistance are also attractive candidates to reduce portal pressure and thus improve portal hypertension. Ang II type 1 blockade is expected to reduce vasoconstriction of portal venules and contractile activity of hepatic stellate cells, resulting in an improved liver perfusion and reduced portal pressure (Bataller and Brenner, 2005). In line with this, we show that losartan, an AT1R blocker, markedly reduced portal pressure in both CCl_4_ and BDL models with portal pressure lowering effect lasted for up to 30 min post-intervention. This rapid effect of losartan reflects its ability to block Ang II/AT1R-mediated intrahepatic vasoconstriction, primarily on portal venules, sinusoidal endothelium, and activated HSCs (Bataller and Brenner, 2005; Herath et al., 2009, 2013). The effects of losartan in reducing portal hypertension is less potent in the BDL model, possibly due to more advanced liver fibrosis in this model compared to the CCl_4_ model (Geerts et al., 2008). AT1R blockers could also be used as anti-fibrotic agents in the fibrotic liver (Yoshiji et al., 2001; Kurikawa et al., 2003; Tox and Steffen, 2006; Nabeshima et al., 2009). However, on the negative side, they are inevitably associated with a number of adverse off-target effects such as systemic hypotension (Croquet et al., 2002; Heller et al., 2003; Tandon et al., 2010), further worsening the condition of cirrhotic patients (Schrier et al., 1988). We therefore sought to investigate whether blockade of MasR or MrgD can reduce the hypotension associated with AT1R blocker treatment. As expected, AT1R blocker losartan caused a large reduction in MAP in both cirrhotic rat models. Although the treatment with MasR or MrgD blockers alone was effective in reducing portal pressure, and increased blood pressure in the BDL model, they failed to increase MAP or reduce portal pressure in any of the groups of losartan-treated rats. This absence of MAP and portal pressure effect of MasR or MrgD blockers when given in combination with losartan may be explained by the potent effect of losartan in our models.

In conclusion, we demonstrate that the alternate RAS plays an important role in controlling portal pressure in cirrhotic but not in non-cirrhotic portal hypertension. The role of MasR, the putative receptor for Ang-(1-7), has been well characterized in cirrhotic portal hypertension; however, this is the first study to report the role of MrgD, the alternate receptor for Ang-(1-7), in splanchnic vasodilatation in cirrhosis. Because a bolus dose of the receptor blockers failed to lower portal pressure to a clinically significant level, the present study warrants further studies in which continuous infusions of the drugs may be expected to produce a clinically significant effect on portal pressure in these models. Nevertheless, we conclude that both MasR and MrgD are potential targets for future therapeutics to treat portal hypertension in cirrhotic patients. We also conclude that the alternate RAS does not contribute to the development of splanchnic vasodilatation in non-cirrhotic portal hypertension.

## Data Availability

The raw data supporting the conclusions of this manuscript will be made available by the authors, without undue reservation, to any qualified researcher.

## Ethics Statement

The experimental procedures in this study were approved by Austin Health animal ethics committee and performed according to the National Health and Medical Research Council (NHMRC) of Australia guidelines for animal experimentation and the principles of the Helsinki declaration.

## Author Contributions

LG and CH designed the experiments. LG performed, acquired, analyzed data, and drafted the manuscript. PA and CH revised and approved the manuscript.

### Conflict of Interest Statement

The authors declare that the research was conducted in the absence of any commercial or financial relationships that could be construed as a potential conflict of interest.
